# Valine-Glutamine Proteins in Plant Responses to Oxygen and Nitric Oxide

**DOI:** 10.3389/fpls.2020.632678

**Published:** 2021-01-25

**Authors:** José León, Beatriz Gayubas, Mari-Cruz Castillo

**Affiliations:** Instituto de Biología Molecular y Celular de Plantas, Consejo Superior de Investigaciones Científicas – Universidad Politécnica de Valencia, Valencia, Spain

**Keywords:** hypoxia, nitric oxide, oxidative stress, oxygen, valine-glutamine proteins, WRKY transcription factors

## Abstract

Multigene families coding for valine-glutamine (VQ) proteins have been identified in all kind of plants but chlorophytes. VQ proteins are transcriptional regulators, which often interact with WRKY transcription factors to regulate gene expression sometimes modulated by reversible phosphorylation. Different VQ-WRKY complexes regulate defense against varied pathogens as well as responses to osmotic stress and extreme temperatures. However, despite these well-known functions, new regulatory activities for VQ proteins are still to be explored. Searching public *Arabidopsis thaliana* transcriptome data for new potential targets of VQ-WRKY regulation allowed us identifying several VQ protein and WRKY factor encoding genes that were differentially expressed in oxygen-related processes such as responses to hypoxia or ozone-triggered oxidative stress. Moreover, some of those were also differentially regulated upon nitric oxide (NO) treatment. These subsets of VQ and WRKY proteins might combine into different VQ-WRKY complexes, thus representing a potential regulatory core of NO-modulated and O_2_-modulated responses. Given the increasing relevance that gasotransmitters are gaining as plant physiology regulators, and particularly considering the key roles exerted by O_2_ and NO in regulating the N-degron pathway-controlled stability of transcription factors, VQ and WRKY proteins could be instrumental in regulating manifold processes in plants.

## Introduction

A group of proteins containing the FxxxVQxxTG motif was first identified in *Arabidopsis thaliana* and named as valine-glutamine (VQ) proteins (Morikawa et al., 2002; Xie et al., 2010; Cheng et al., 2012). Up to 34 VQ proteins have been identified in *A. thaliana* (Cheng et al., 2012). The analysis of the regulatory activity of Arabidopsis VQ proteins revealed that all but five exhibited transcriptional regulatory activity, 17 activating and 12 repressing gene transcription (Li et al., 2014a). The integrity of the VQ motif seems to be essential for VQ4/MVQ1 and VQ29 regulatory activities (Li et al., 2014b; Weyhe et al., 2014) likely because their regulation often relies on the interaction with WRKY transcription factors (Cheng et al., 2012). The functional interaction of a subset of Arabidopsis VQ proteins, comprising 10 members, with WRKY transcription is modulated by reversible phosphorylation catalyzed by MAP kinases (Pecher et al., 2014; Weyhe et al., 2014). After the initial identification in Arabidopsis, VQ protein families have been also identified in a large number of plants, including rice, soybean, grapevine, Chinese cabbage, maize, banana, bamboo, strawberry, apple, tea plant, Eucalyptus, tobacco, chick pea, and alfalfa (Kim et al., 2013; Li et al., 2014a; Wang et al., 2014, 2015a, 2017; Zhang et al., 2015; Song et al., 2016; Ye et al., 2016; Zhou et al., 2016; Dong et al., 2018; Guo et al., 2018; Zhong et al., 2018; Garrido-Gala et al., 2019; Yan et al., 2019; Ling et al., 2020; Liu et al., 2020). The size of the VQ proteomes varies ranging from seven members identified in *Selaginella moellendorffii* to 74 in *Glycine max* (Jiang et al., 2018). While VQ proteins were initially thought to be plant specific proteins (Jing and Lin, 2015), recent studies on diverse genomes concluded that VQ proteins are also present in bacteria, fungi, and lower animals but not in algae (Jiang et al., 2018). The regulatory functions exerted by VQ proteins are manifold, and include defense against biotic (Xie et al., 2010; Lai et al., 2011; Wang et al., 2015b; Jiang and Yu, 2016; Chen et al., 2018; Yan et al., 2018) and abiotic stresses (Perruc et al., 2004; Hu et al., 2013b; Song et al., 2016; Cheng et al., 2020), and plant growth (Wang et al., 2010; Li et al., 2014b; Lei et al., 2017, 2018; Pan et al., 2018). Nevertheless, most of the plant VQ protein functions remain unknown.

## VQ Proteins Regulation of Development

Valine-glutamine proteins regulate developmental processes such as pollen or seed germination, plant size, photomorphogenesis, and leaf senescence. IKU1/VQ14 was characterized as a component of the so-called HAIKU pathway controlling the early growth phase of the seed endosperm (Garcia et al., 2003). *iku1* mutant seeds were small and showed reduced endosperm growth (Wang et al., 2010). Chloroplast targeted VQ8 also plays a role in regulating growth as *vq8-1* mutant displayed stunted-growth and pale-green leaves throughout the entire life cycle (Cheng et al., 2012). However, the over-expression of *VQ17*, *VQ18*, or *VQ22*, also led to highly stunted transgenic plants (Cheng et al., 2012), thus suggesting VQ proteins might promote or repress plant growth. Moreover, the over-expression of *VQ29* delayed flowering time without altering vegetative growth (Cheng et al., 2012), but the expression of Arabidopsis *VQ21* resulted in dwarfed and late-flowering plants (Gargul et al., 2015), thus suggesting *VQ* gene-specific functions may also uncouple different developmental processes. In addition, the heterologous overexpression of several soybean *VQ* genes in Arabidopsis led to altered leaf morphology, flowering, and seed setting (Zhou et al., 2016), thus indicating that developmental regulatory roles of VQ proteins are likely conserved across species. Moreover, the Arabidopsis *vq29* mutant exhibited decreased hypocotyl elongation under low-intensity far-red and white light (Li et al., 2014b), thus pointing to VQ29 as a negative regulator of photomorphogenesis (Li et al., 2014b).

**Figure 1:**
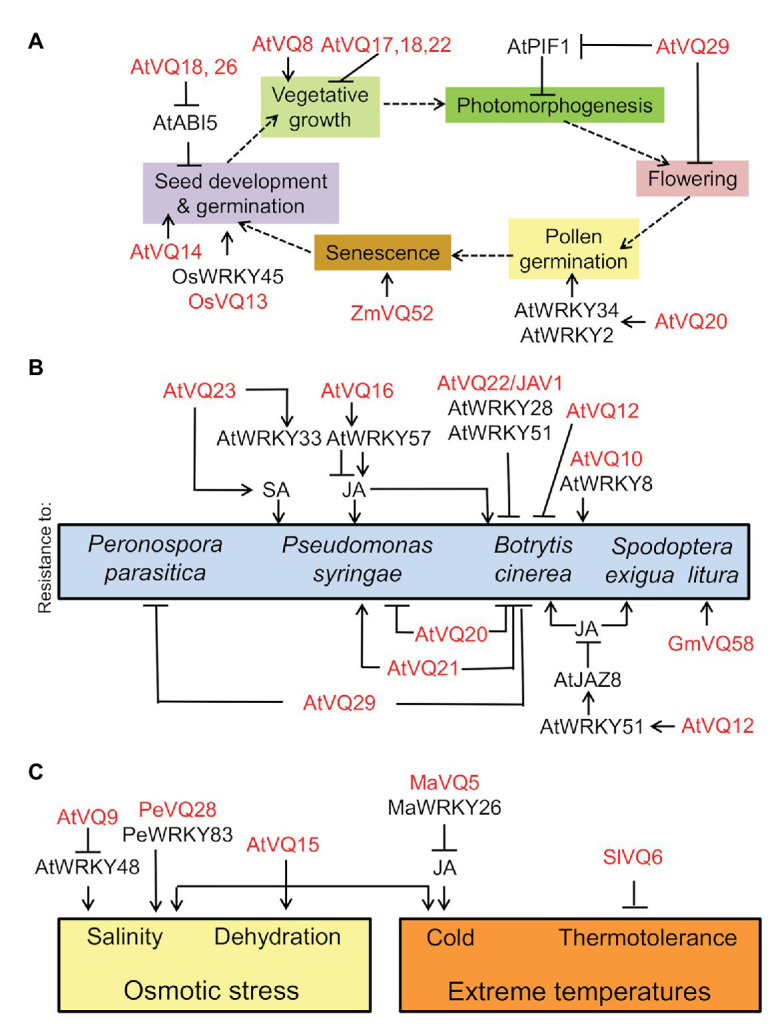
Involvement of valine-glutamine (VQ) proteins and WRKY transcription factors in developmental processes throughout plant life cycle **(A)**, in resistance of plants against biotrophic and necrotrophic pathogens as well as insects **(B)**, and in responses to abiotic stress factors **(C)**. VQ and WRKY proteins from different plants (At, *Arabidopsis thaliana*; Gm, *Glycine max*; Ma, Musa acuminate; Os, *Oryza sativa*; Pe, *Phyllostachys edulis*; Sl, *Solanum lycopersicum*; Zm, *Zea mays*) regulate positively (arrow lines) or negatively (blunt-ended lines).

VQ20 regulates pollen development through its VQ motif by acting together with WRKY2 and WKRY34 in plant male gametogenesis (Lei et al., 2017) through the negative regulation of the expression of *MYB97*, *MYB101*, and *MYB120* genes (Lei et al., 2018). Some of the development-related processes regulated by VQs are linked to phytohormone action. OsVQ13 positively regulated jasmonic acid (JA) signaling by activating the OsMPK6-OsWRKY45 signaling pathway that regulates grain size and resistance to Xanthomonas in rice (Uji et al., 2019). On the other hand, Arabidopsis seed germination seems to be controlled through the negative regulation exerted by VQ18 and VQ26 on ABI5 transcription factor-mediated ABA signaling (Pan et al., 2018). However, neither seed dormancy or leaf senescence nor ABA-regulated drought tolerance were significantly regulated by VQ18 and VQ26 (Pan et al., 2018), thus pointing to highly specific regulation. Leaf senescence is another developmental process potentially regulated by VQ proteins. The overexpression of maize *ZmVQ52* in Arabidopsis accelerated premature leaf senescence (Yu et al., 2019). Figure 1A summarizes what has been reported on the involvement of VQ proteins and WRKY transcription factors in regulating different processes throughout plant life cycle.

## VQ Proteins in Defense Against Pathogens and Pests

Reports during the last decade supported the function of VQ proteins as relevant regulators of plant defense against pathogens and pests. The first report involving VQ proteins in defense against pathogens identified SIB1/VQ23 as an activator of JA-dependent salicylic acid (SA)-triggered resistance to *Pseudomona syringae* (Xie et al., 2010), and together with SIB2/VQ16, WRKY33, and WRKY57 also activated resistance to the necrotrophic pathogen *Botrytis cinerea* through the Jasmonate-zim-domain 1 (JAZ1) and 5 (JAZ5) proteins (Lai et al., 2011; Jiang and Yu, 2016). By contrast, VQ20 acted as a negative regulator of resistance to both biotrophic and necrotrophic pathogens (Cheng et al., 2012). MKS1/VQ21, in turn, positively regulated SA-mediated defense against biotrophic pathogens but it plays a negative role in JA-regulated defense against necrotrophic pathogens (Andreasson et al., 2005; Petersen et al., 2010). Regulation of resistance to Botrytis by VQ21 also requires the interaction with WRKY33 at the VQ motif domain (Petersen et al., 2010). Different VQ-WRKY complexes not only allow discriminating between different pathogens but also between defense and development. Silencing the *JAV1/VQ22* gene significantly enhanced JA-regulated defense responses against necrotrophic pathogens and herbivorous insects by forming complexes with WRKY28 and WRKY51 but did not severely alter JA-mediated development (Hu et al., 2013a). Mutant or transgenic plants with double loss-of-function and gain-of-function in *VQ12* and *VQ29* genes were resistant and susceptible, respectively, to *B. cinerea* (Wang et al., 2015b), thus supporting the role of these VQ proteins as negative regulators of defense against this pathogen. Besides, the inactivation of *VQ29* gene significantly increased susceptibility to *Peronospora parasitica* during the late stages of infection likely due to the inability to restrict the penetration and development of the oomycete (Le Berre et al., 2017). Other combinations of VQ proteins with WRKY transcription factors are also involved in modulating resistance to Botrytis. VQ10 physically interacts with WRKY8 and positively regulates plant basal resistance (Chen et al., 2018). On the other hand, strawberry homologs of Arabidopsis VQ defense proteins were all regulated in response to the ascomycete fungus *Colletotrichum acutatum* infection, causing anthracnose disease (Garrido-Gala et al., 2019). In tobacco, half of the 59 identified VQ protein encoding genes were significantly induced in response to *Ralstonia solanacearum* infection (Liu et al., 2020), thus supporting the potential extensive roles of VQ proteins in tobacco defense against this pathogen.

The complex roles of *VQ* genes in plant defense responses are likely due to their ability to interact with multiple WRKY proteins that in Arabidopsis were modulated through MAP Kinase-mediated phosphorylation and further degradation of VQ proteins (Pecher et al., 2014; Weyhe et al., 2014). Similar regulatory mechanisms seem to be operational also in rice (Li et al., 2014a) and Cucurbitaceae plants (Jiao et al., 2018), having an impact on regulating resistance to powdery mildew. Altogether, the involvement of VQ proteins in regulating defense against different pathogens is complex and gene-specific, likely occurring through combinatorial mechanisms involving other partners as well as functional interaction with diverse hormone-regulated pathways. These regulatory mechanisms seem to be also functional in plants attacked by insects. Injury rapidly triggers calcium influxes, calmodulin-dependent phosphorylation of JAV1/VQ22, dismantling of JAV1-JAZ8-WRKY51 complex, and activation of JA biosynthesis for plant defense (Yan et al., 2018). JAV1-associated Ubiquitin Ligase1 (JUL1) is the RING-type E3 ubiquitin ligase leading JAV1 to proteasomal degradation (Ali et al., 2019). In soybean, the down-regulation of *GmVQ58* confers resistance to the common cutworm *Spodoptera litura* Fabricius (Li et al., 2020).

Summarizing, specific subsets of VQ proteins may regulate different pathosystems with process specificity through a complex network of interactions with WRKY transcription factors (Figure 1B). The distinct resulting complexes are often regulated through post-translational modifications (PTMs), with reversible phosphorylation being the best characterized.

## VQ Proteins and Abiotic Stress

Most of the information on plant VQ protein functions in responses to abiotic stress is related to osmotic stress. *AtCaMBP25/VQ15* expression is induced in Arabidopsis seedlings exposed to dehydration, low temperature, or high salinity (Perruc et al., 2004). Transgenic plants overexpressing *AtCaMBP25* exhibited increased sensitivity to both ionic and non-ionic osmotic stress during seed germination and seedling growth (Perruc et al., 2004). VQ9 protein acted as a repressor of the WRKY8 factor to maintain an appropriate balance of WRKY8-mediated signaling pathways and the onset of salinity stress tolerance (Hu et al., 2013b). In bamboo, PeVQ28 and WRKY83 interacted in the nucleus, and the over-expression of *PeVQ28* in Arabidopsis led to increased resistance to salt stress and enhanced sensitivity to ABA (Cheng et al., 2020). Besides responses to osmotic stress, VQ proteins regulate responses to extreme temperatures. In banana fruits, MaVQ5 might act as a repressor of MaWRKY26 in activating JA biosynthesis in response to cold stress (Ye et al., 2016). On the other hand, ectopically overexpressed tomato *SlVQ6* in Arabidopsis plants decreased thermotolerance (Ding et al., 2019). The main regulatory roles exerted by VQ proteins on plant responses to abiotic stress factors are summarized in Figure 1C.

## Molecular Oxygen and Nitric Oxide Regulation on the Arabidopsis VQ Protein Family

An important though still mostly unexplored feature of VQ proteins is their subcellular localization. An *in silico* analysis of subcellular localization for Arabidopsis VQ proteins points to predominantly nuclear localization. However, VQ1 and VQ10 are potentially localized both in nuclei and cytoplasm, and others (VQ3, VQ8, VQ12, VQ19, VQ20, VQ23/SIB1, VQ16/SIB2, and VQ31) both in nuclei and chloroplasts. The nucleus/chloroplast alternative localizations of some VQ proteins may be potentially involved in plastid-nucleus retrograde and anterograde signaling (Unal et al., 2020). On the other hand, nucleo-cytoplasmic shuttling of regulatory proteins is often modulated by PTMs. Although phosphorylation of VQ proteins has been documented (Pecher et al., 2014; Weyhe et al., 2014), many other still unknown PTMs might regulate the subcellular localization and dynamics of VQ proteins. All VQ proteins but VQ3 might be potentially ubiquitinated and acetylated in K residues. In turn, only some are predicted to be sumoylated in K, palmitoylated or S-nitrosylated in C residues, and nitrated in Y. VQ6, VQ7, VQ8, VQ9, and VQ12 are predicted to be both S-nitrosylated and palmitoylated in the same C residue at the N-terminus of the proteins, thus suggesting both PTMs compete for the same sites. These alternative PTMs may be critical to determine the subcellular localization and transcriptional activity of these VQ proteins. More work will be needed to support this hypothesis and to clarify whether PTMs can determine the fate, localization, and function of VQ proteins.

**Figure 2:**
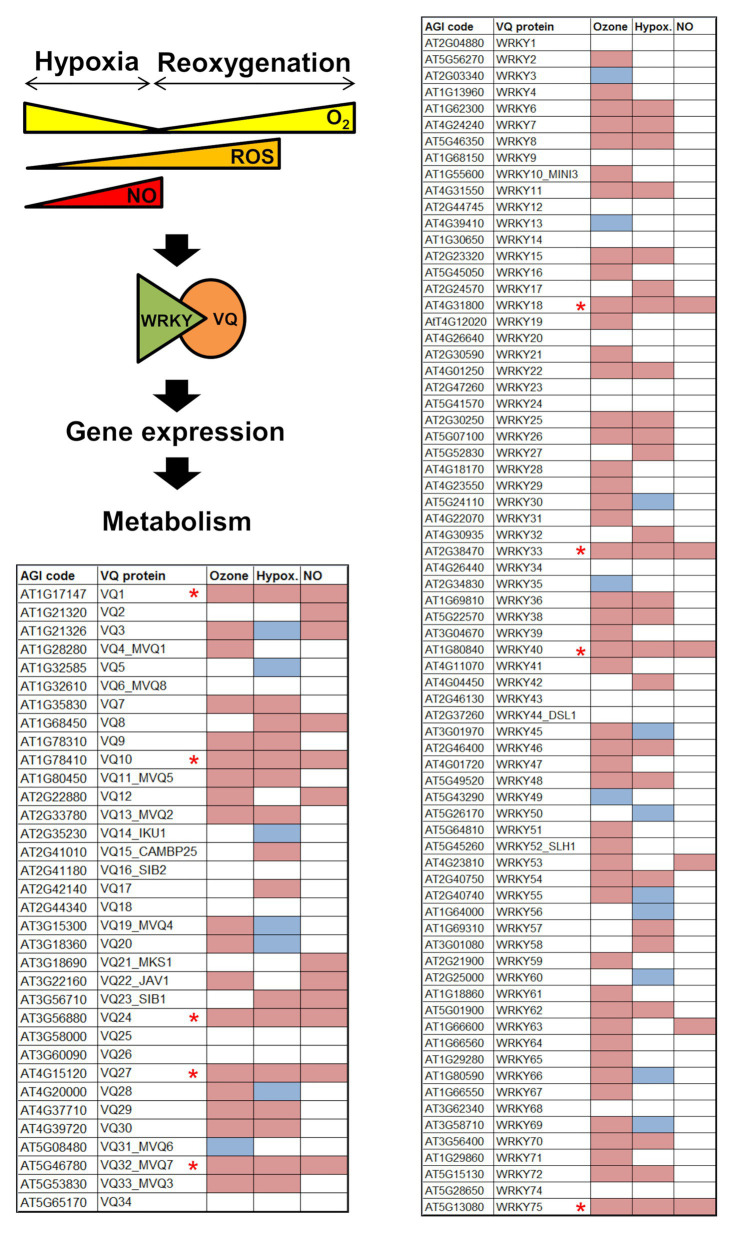
Regulation of Arabidopsis VQ proteins and WRKY transcription factors encoding genes in response to ozone-triggered Reactive Oxygen Species (ROS) production (Ozone), hypoxia (Hypox.), and nitric oxide (NO) treatment (NO). Upregulated (magenta) and down-regulated (blue) transcripts identified in ozone-treated Col-0 plants (Xu et al., 2015), in response to hypoxia and re-oxygenation after hypoxia (Lee and Bailey-Serres, 2019), and in plants exposed to a NO pulse (Castillo et al., 2018; León et al., 2020). Genes marked with red asterisks are upregulated by ozone, hypoxia and NO.

Analysis of public repositories of transcriptome data allowed proposing processes potentially regulated by VQ proteins. Gene Ontology categories enrichment suggests that a significant number of Arabidopsis *VQ* genes were upregulated under ozone-triggered oxidative stress and differentially expressed in response to low oxygen availability. Molecular oxygen and their metabolites, mainly reactive oxygen species (ROS), have gained relevance lately as key signaling molecules in plant development and responses to stress (Van Breusegem and Dat, 2006; Suzuki etal., 2012; van Dongen and Liacausi et al., 2015; Choudhary et al., 2020; Dogra and Kim, 2020; Fichman and Mittler, 2020; Weits et al., 2020). Ozone has been used as a tool to study the role of ROS in cell death and defense signaling as well as in regulating gene expression (Vainonen and Kangasjärvi, 2015). The analysis of the differentially expressed transcriptome in ozone-treated Arabidopsis plants (Xu et al., 2015) allowed identifying that 56% of the *VQ* genes (19 out of 34) and 64% of the *WRKY* genes (48 out of 75) were upregulated by ozone (Figure 2). These data suggest that ozone seems to extensively activate *VQ* and *WRKY* genes, thus suggesting that distinct VQ-WRKY complexes might regulate plant responses to ROS.

Plants usually grow and develop in 21% O_2_ normoxic environment. However, plants are sometimes exposed to hypoxic conditions and do not have specific O_2_ transporters, like hemoglobin in animals, which allow transport between different plant organs or tissues. Instead, plants rely on diffusion between cells or in passive transport through vascular tissue as oxygen transport mechanisms (Armstrong et al., 2006). Importantly, plants contain tissues and organs such as root internal cells, apical meristems, or fruits, where different physical or metabolic barriers preclude oxygen diffusion, thus causing hypoxic niches (Considine et al., 2017; Weits et al., 2019; Labandera et al., 2020; Mira et al., 2020). On the other hand, hypoxia may be imposed by heavy rainfall and the subsequent flooding of lands, which maintain plants transiently submerged or waterlogged (Voesenek and Bailey-Serres, 2015). When water recedes, hypoxic plants undergo a rapid re-oxygenation that lead to the production and metabolism of ROS and NO. A combined analysis of transcriptome data on exogenous NO treatment (Castillo et al., 2018; León et al., 2020) and in response to hypoxia and re-oxygenation after hypoxia (Lee and Bailey-Serres, 2019) allows identifying a cluster of NO-regulated VQ protein encoding genes that were upregulated and downregulated by hypoxia and re-oxygenation after hypoxia, respectively (Figure 2). A similar analysis focusing on *WRKY* genes allowed also identifying a cluster of four *WRKY* genes that were upregulated by hypoxia and NO, and downregulated upon re-oxygenation (Figure 2). Five genes of that VQ cluster (*VQ1*, *VQ10*, *VQ24*, *VQ27*, and *VQ32*) and the four WRKY genes (*WRKY18*, *WRKY33*, *WRKY40*, and *WRKY75*) were also upregulated under ozone treatment (Figure 2). Altogether, these data suggest that some VQ proteins, likely in association to some WRKY transcription factors, may play relevant roles in responses to changes in oxygen availability, ROS and NO in plants. VQ-WRKY regulatory actions might be exerted in a combinatorial way, so that the elucidation of the dynamics and relative VQ-WRKY affinities will be essential to better know the mode of action of these regulatory complexes.

Nitric oxide might be the potential link between VQ-WRKY modules and the responses to oxidative stress, hypoxia, and other NO-regulated processes. Plants accumulate NO in response to ozone (Mahalingam et al., 2006; Ahlfors et al., 2009; Pasqualini et al., 2012; Bison et al., 2018; Li et al., 2018), and because of the mitochondrial electron chain using nitrite as electron acceptor also under oxygen limiting conditions (Gupta et al., 2018). The subset of *VQ* and *WRKY* genes that are upregulated in plants under oxidative stress, hypoxia, and treatment with NO may represent components of potential VQ-WRKY core complexes controlling downstream gene expression and metabolic alterations in a wide range of physiological processes (Figure 2). Interactions between VQ1 and VQ10 with WRKY33, VQ24 with WRKY75, WRKY18 with WRKY33 and WRKY40, have been all reported (Xu et al., 2006; Pandey et al., 2010; Arabidopsis Interactome Mapping Consortium, 2011; Cheng et al., 2012; Birkenbihl et al., 2017; Abeysinghe et al., 2019) in stress-related responses. Developmental programs such as leaf senescence are also regulated by NO and ROS, and they represent potential new targets for VQ-WRKY protein regulation. The relationship of the senescence process and the production of NO is somehow controversial as both positive or negative correlation has been reported depending upon the organ or being natural or dark-induced (Mishina et al., 2007; Ma et al., 2010; Niu and Guo, 2012; Liu and Guo, 2013; Du et al., 2014; Bruand and Meilhoc, 2019). Linked to ROS and NO action, ZmVQ52 associated to WRKY proteins regulate leaf senescence in maize (Yu et al., 2019). Moreover, around 32% of the *VQ* genes and more than half of the *WRKY* genes were upregulated in Arabidopsis senescing leaves (Schmid et al., 2005). Some of these genes were also differentially expressed in leaves in the transition from mature to senescent leaves (Woo et al., 2016).

## Concluding Remarks and Perspectives

Proteins containing the VQ motif have been studied during the last 20years with increasing attention being gained during the last decade. Despite the regulatory functions of some VQ proteins have been characterized in development and stress responses, most of the processes regulated by VQ proteins remain unknown. Importantly, the modes of action by which VQ proteins regulate these processes are still incompletely understood though their functional associations to WRKY factors seem to be important. Nevertheless, the identification of the VQ-WRKY complexes and the characterization of their affinities in different processes remain yet to be analyzed. Furthermore, the functional connection between VQ proteins and gasotransmitters such as O_2_ and NO opens up multiple developmental and stress-related processes potentially regulated by VQ proteins. Among them, hypoxia-triggered responses and subsequent re-oxygenation recovery are very relevant to modulate the tolerance of plants to submergence and waterlogging in flooded lands, a stressful condition becoming increasingly common in the context of climate change. On the other hand, some VQ proteins and their WRKY partners are also regulated by NO likely through NO-triggered PTMs that remain yet to be identified. Future questions that need to be also addressed include the elucidation of new WRKY-independent VQ protein regulatory functions that will benefit from the combination of genetic and omics approaches.

## Author Contributions

JL wrote the article, conceived the project and supervised co-authors draft writing. BG and M-CC had an equal contribution in collecting information. All authors contributed to the article and approved the submitted version.

### Conflict of Interest

The authors declare that the research was conducted in the absence of any commercial or financial relationships that could be construed as a potential conflict of interest.
